# The Influence of Playing a Non-Reward Game on Motor Ability and Executive Function in Parkinson’s Disease

**DOI:** 10.3233/BEN-2011-0353

**Published:** 2012-01-20

**Authors:** Alisson Menezes Araújo Lima, Fabiana de Campos Cordeiro Hirata, Gabriela Sales de Bruin, Rosa Maria Salani Mota, Veralice Meireles Sales de Bruin

**Affiliations:** Department of MedicineFederal University of CearáCearáBrazil

**Keywords:** Parkinson, game, dopamine, memory, depression

## Abstract

The aim of this study is to evaluate the acute effect of playing games on executive function and motor ability in Parkinson's disease (PD). Consecutive cases with PD were studied with the Unified Parkinson Disease Rating Scale (UPDRS), Mini-Mental State examination (MMSE), Beck Depression Inventory (BDI), Stroop test, finger tapping and 14-meter walk test. After randomization, patients performed a game of dominoes and were tested before and after experiment being further categorized as control, winners or non-winners. Forty patients, 27 male (67.5%), aged 48 to 84 years (63.2 ± 8.5), Hoehn & Yahr I to III were included. Twenty-eight (70%) presented depressive symptoms (BDI > 10). Groups (Control N = 13; Winners = 14 and Non-winners = 13) were not different regarding age, disease duration, age at onset, BMI, MMSE scores, depressive symptoms, levodopa dose, and previous practice of games. Winners presented significantly better results on executive function (Stroop test, *p* = 0.002) and on motor activity (Finger tapping, *p* = 0.01). Non-winners showed a trend of better performance in the 14-meter-walk test. This study shows that the practice of a non-reward game acutely improved memory and motor skills in PD. Our results suggest a role for the reward system in the modulation of the dopaminergic function of the basal ganglia in these patients.

## 1. Introduction

Parkinson's disease (PD) is caused by a nigrostriatal dopamine deficiency and clinically presents with the classic triad of resting tremor, rigidity, and bradykinesia. Non-motor symptoms such as mild cognitive or psychiatric impairments such as bradyphrenia and depression have been recognized as important issues associated with worse quality of life and increased mortality [1]. It has been suggested that PD patients may present two components of cognitive dysfunction: a more general profile of subcortical dementia and/or a pattern suggesting more specific cerebral pre-frontal involvement. The first type of cognitive dysfunction has been named subcortical dementia and refers to a clinical syndrome associated with structural neocortical/subcortical changes in the brain, meaning frontal, parietal, limbic, and temporal lobes, as well as midbrain pathological involvement. Slowing of cognition, difficulty with complex intellectual tasks such as strategy generation and problem solving, visuospatial abnormalities, changes in personality and disturbances of mood found in conjunction with relatively preserved language and memory functions would be more common in the sub-cortical cognitive dysfunction type. The second and more selective pre-frontal type of cognitive dysfunction, considered a result of dysfunction in neuronal loops connecting the prefrontal cortex and basal ganglia, would manifest with impairment of neuropsychological tests sensitive for frontal lobe function such as those involving episodic and working memory. These findings are of clinical relevance since cognitive dysfunction has been demonstrated in over 50%

ISSN 0953-4180/12/$27.50 © 2012 – IOS Press and the authors. All rights reserved of cases of PD and the impairment of executive/motor component is reported to be present in over 80% of cases [2].

It has been shown that brain activation correlates with expected reward and also that subcortical dopaminergic structures such as striatum are involved in reward-related processes [3,4]. This is a complex subject since previous abilities and practice effect [5,6], emotions [7] and even intentional stance meaning on line mentalizing [8] have been shown to alter brain function. Previous studies have shown that impulse control disorders such as pathologic gambling, excessive spending and hypersexuality found in Parkinson's disease (PD) [1,2] might be connected to an altered dopaminergic reward system [8,9]. Moreover, non-reward games have been shown to modify cognitive function. Playing an action video game improved and modified gender differences in spatial attention [9]. Video-game playing is capable of altering a range of visual skills [10]. The game of Bingo has been associated with cognitive stimulation and improved performance in neurocognitive tests better than daily physical activity [11].

We hypothesized that PD patients are susceptible to brain activation and that this activation may be associated with non-reward gaming. The aim of this study was to test whether playing a non-reward game of dominoes acutely modifies motor abilities and executive function in PD patients.

## 2. Methods

## 3. Study design

This was an experimental study, involving a sample of PD patients, Hoehn and Yahr I to III, who were consecutively recruited from an outpatient hospital based clinic. MMSE score above 20 was required to enter the study [12]. None of the subjects were using direct sleep promoting substances such as benzodiazepines or antidepressants. No medications had been altered during the three months preceding the study. Cases with supranuclear gaze palsy, autonomic dysfunction, cerebellar signs, hand apraxia and hallucinations were excluded. Also, cases with cancer, drug or alcohol abuse, severe neurologic, renal, hepatic, lung or cardiac diseases were excluded. All data were collected at the same time of the day between 8 and 9 A.M (30 minutes tolerance). The protocol was approved by the local Research Ethics Committee and written informed consent was obtained in all cases.

### *3.1. Subject characteristics*

We studied 40 patients with clinical diagnosis of PD aged from 48 to 84 years (mean 63.20 $\pm$ 8.55), 27 (67.5%) of male gender. Disease duration varied from 1 to 25 years (mean 6.55 $\pm$ 5.03). All patients were clinically diagnosed as PD, were responsive to levodopa therapy and were on stable doses of levodopa or other dopamine agonists in the last three months. Levodopa dose varied from 150 to 1250 mg (mean 653.75 $\pm$ 283.1). Body mass index varied from 19.8 to 32 (mean 24.5 $\pm$ 2.5).

### *3.2. Experimental procedure*

Assessment included the Unified Parkinson's Disease Rating Scale (UPDRS), Folstein Mini-Mental State Examination (MMSE), Beck Depressive Inventory (BDI) [13], Finger Tapping Test [14], 14-meter walking test (14-MWT) and the Stroop test (Phase 3, time in seconds measured) [15]. Measures were taken before and immediately after experiment. Protocol consisted of playing a game of dominoes, starting at 8:00 AM (with 30 minutes of tolerance) with one previous training session. Training and playing lasted about 40 minutes. Patients were randomized for playing or not and those that did not play were asked to sit and while sitting, they read a magazine or watched a movie. Experiment was started within 15-30 minutes after the first daily dose of levodopa. All measures were taken before and after playing the game. Examiners were blind to which group patients belonged.

### *3.3. Statistical analysis*

Data are expressed as mean, standard deviation, standard error and percentage values. Kolmogorov-Smirnoff test was used for evaluation of the residual normality, the Levene test for evaluation of equality of variance and the exact Fisher test to compare groups. After experiment, patients were classified as control, winners and non-winners. ANOVA was used for between groups comparison. A linear regression analyzes was done to evaluate associations between variables. Factorial analyses for repeated measures were performed to study the effects of the practice of the game on motor activity and memory test. The factorial model was adjusted utilizing co-variables selected among those that presented $p < 0.10$ in the regression analyzes. The level of significance was of 95%. Using the Bonferroni corrections, the level of significance was at $p < 0.02$. All variables were submitted to a package of the SPSS for Windows.

Table 1
Clinical characteristics of the study population grouped as control, winners and non-winners

| | Control (N = 13) | Non-winners (N = 13) | Winners (n = 14) | ANOVA |
|---|---|---|---|---|
| Gender (M/F) | 10/3 | 7/6 | 10/4 | |
| Age | | | | |
| Range | 48–72 | 55–84 | 51–73 | P = 0.10 |
| Mean ± SD | 59.92 ± 9.01 | 67 ± 8.34 | 62.71 ± 7.38 | |
| Age at disease onset | | | | |
| Range | 43–69 | 42–80 | 41–67 | P = 0.15 |
| Mean ± SD | 52.69 ± 8.62 | 59.31 ± 10.53 | 58.29 ± 8.31 | |
| Disease duration | | | | |
| Range | 2–16 | 1–25 | 1–10 | P = 0.13 |
| Mean ± SD | 7.23 ± 4.42 | 8.15 ± 7.01 | 4.43 ± 2.14 | |
| Levodopa dose (mg/day) | | | | |
| Range | 150–1000 | 500–1250 | 150–1250 | P = 0.22 |
| Mean ± SD | 542.31 ± 255.84 | 721.15 ± 240.19 | 694.64 ± 328.63 | |
| BMI | | | | |
| Range | 19.83–27.05 | 20.03–30.00 | 22.86–32.05 | P = 0.11 |
| Mean ± SD | 23.45 ± 1.92 | 24.79 ± 2.87 | 25.47 ± 2.48 | |
| UPDRS | | | | |
| Range | 0–35 | 1–30 | 0–26 | P = 0.76 |
| Mean ± SD | 11.38 ± 10.40 | 13.23 ± 8.91 | 10.79 ± 7.30 | |
| BDI | | | | |
| Range | 0–24 | 1–27 | 0–29 | P = 0.95 |
| Mean ± SD | 13.23 ± 7.86 | 13.31 ± 8.01 | 12.43 ± 7.93 | |

Abbreviations: BMI = Body Mass Index; UPDRS = Unified Parkinson's disease Rating Scale, BDI = Beck Depressive Inventory.

Table 2
Past history of alcohol consumption, smoking, use of dopaminergic agonist, family history of Parkinson's disease and previous practice of games of patients grouped as controls or experiment (winners or non-winners)

| Variables | Controls N (%) | Winners N (%) | Non-winners N (%) | Fisher Test |
|---|---|---|---|---|
| Heavy drinking | 6 (46.1%) | 5 (38.5%) | 7 (50%) | $^{a}p = 1.0$ <br> $^{b}p = 0.74$ |
| Smokers | 3 (23.1%) | 4 (30.8%) | 7 (50%) | $^{a}p = 0.31$ <br> $^{b}p = 0.44$ |
| Use of dopamine agonists | 6 (46.1%) | 6 (46.1%) | 5 (35.7%) | $^{a}p = 0.78$ <br> $^{b}p = 0.70$ |
| Family history of PD | 6 (46.1%) | 5 (38.5%) | 5 (35.7%) | $^{a}p = 0.73$ <br> $^{b}p = 1.0$ |
| Previous habit of games | 5 (38.5%) | 5 (38.5%) | 8 (57.1%) | $^{a}p = 0.73$ <br> $^{b}p = 0.45$ |

$^{a}$ = Control versus non-winners and $^{b}$ control versus winners.

## 4. Results

After initial assessment, patients ($n = 40$) were randomized to play dominoes (Experiment, $n = 27$) or to remain seated reading or watching television (control, $n = 13$). Cases in the group that played were further classified in winners ($n = 14$) or non-winners ($n = 14$). Clinical and demographic features of the study population are depicted in Table 1. Age, age at disease onset, disease duration, levodopa dose, BMI, motor disability, as evaluated by the UPDRS, and BDI-21 scores were not different among groups (Table 1). Depressive symptoms (BDI > 10) were found in over 60% of cases; BDI-21 scores varied from 0 to 29, no gender differences being found (male = 12.7 ± 7.3; female = 13.4 ± 8.8; $p = 0.78$). Eighteen patients (45%) related frequent consumption of alcoholic beverages. The habit of smoking was present in 14 cases (35%) and the use of dopamine agonists was registered in 17 individuals (42.5%). Eighteen individuals described previous habits of playing games that included dominoes and pack of cards. Alcohol consumption, smoking, use of dopamine agonists and previous practice of game were not different among groups (Table 2).

Table 3
Linear correlation between test values, clinical-demographic data and scores of behavior scales

| | | Stroop Test | | Finger Tapping | | 14-MWT (sec) | |
|---|---|---|---|---|---|---|---|
| | N | R | P | R | P | r | P |
| Baseline evaluation | | | | | | | |
| Age at evaluation | 40 | 0.2949 | 0.06 | −0.2320 | 0.14 | 0.4012 | 0.01* |
| BMI | 40 | 0.0365 | 0.82 | −0.0365 | 0.82 | 0.1239 | 0.44 |
| Income | 40 | 0.1266 | 0.43 | 0.0462 | 0.77 | −0.0508 | 0.75 |
| Age at disease onset | 40 | 0.1248 | 0.44 | −0.1241 | 0.44 | 0.3456 | 0.02* |
| Duration of illness | 40 | 0.3164 | 0.04* | −0.1795 | 0.26 | 0.0262 | 0.87 |
| Dose of levodopa | 40 | 0.0926 | 0.56 | 0.0223 | 0.89 | 0.3801 | 0.01* |
| BDI | 40 | 0.2060 | 0.20 | −0.3715 | 0.01* | 0.4234 | 0.005** |
| UPDRS part III | 40 | 0.0762 | 0.64 | −0.2751 | 0.08 | 0.4783 | 0.001** |
| Post-experimental evaluation | | | | | | | |
| Age at evaluation | 40 | 0.3004 | 0.05 | −0.1364 | 0.40 | 0.3438 | 0.02* |
| BMI | 40 | −0.0234 | 0.88 | 0.0702 | 0.66 | 0.0647 | 0.69 |
| Income | 40 | 0.1530 | 0.34 | 0.0434 | 0.79 | −0.0437 | 0.78 |
| Age at disease onset | 40 | 0.1968 | 0.22 | −0.0246 | 0.88 | 0.2899 | 0.06 |
| Duration of illness | 40 | 0.1781 | 0.27 | −0.1954 | 0.22 | 0.0306 | 0.85 |
| Dose of levodopa | 40 | 0.0734 | 0.65 | 0.0130 | 0.93 | 0.3244 | 0.04* |
| BDI | 40 | 0.2786 | 0.08 | −0.3882 | 0.01* | 0.4644 | 0.002** |
| UPDRS part III | 40 | 0.1486 | 0.36 | −0.3636 | 0.02* | 0.5485 | 0.0002** |

Abbreviatons: BMI = Body Mass Index; BDI = Beck Depression Inventory; UPDRS = Unified Parkinson's Disease Rating Scale; 14-MWT = 14-meter Walk Test.
$^{*}p < 0.05$; $^{**}p < 0.01$.

Descriptive data of the Stroop Test, Finger Tapping and 14-MWT of patients grouped as controls or experiment (winners or non-winners) are given in Table 3. After experimentation, controls did not improve executive function, and motor ability as evaluated by the 14-MWT and finger tapping. Only winners showed improvement of executive function as evaluated by the Stroop test and finger tapping. Non-Winners improved motor ability as evaluated by the 14-MWT (Table 4). Several correlations were found between clinical data and test values (Table 4). Older age at evaluation and at disease onset were correlated to greater time spent in the 14-MWT. After experimentation, age correlation to 14-MWT was kept while age at disease onset showed only a trend. Longer duration of illness was correlated to increased time spent in the Stroop test and this correlation was not maintained after experiment. Scores of the BDI were correlated to worse performance in the finger tapping and 14-MWT and those were maintained after the experimental procedure. Higher scores of the UPDRS III, meaning impairment of motor function, were correlated to decreased finger tapping and increased time in the 14-MWT and this was maintained after experimental procedure.

After experimentation, both winners and non-winners groups showed improvement of executive function as evaluated by the Stroop test. After comparisons between the two experimental groups, winners showed better performance of the Stroop test and finger tapping than controls and non-winners. Interestingly, non-winners performed better than controls and winners in the 14-MWT. Table 4 depicts results before and after Bonferroni corrections. Executive function remained better in winners after adjusting for age and disease duration ($p = 0.002$). Finger tapping remained better for winners after controlling for age at disease onset, use of dopamine agonists and UPDRS evaluation ($p = 0.01$). After adjusting for age, age at disease onset, use of dopamine agonists and UPDRS evaluation, non-winners showed a trend for better performance in the walking test ($p = 0.02$).

## 5. Discussion

This study shows that the act of playing a game improves executive function and motor skills of patients with PD. Winners showed better performance confirming the influence of the reward system on mechanisms of selective attention and motility. In this experiment, we did not use gambling to avoid unwanted practices and we showed that the simple practice of playing games is beneficial.

Our results show that patients that played generally performed better in motor skills. Finger tapping was better in winners, and non-winners showed a trend for a better walking time. Studies have shown that expected reward and risk have been correlated to cortical regions

Table 4
Results of the Stroop Test, Finger Tapping Test and 14-meter Walk Test, at baseline and post experiment, in winners and non-winners, before and after Bonferroni's corrections

| Group | Experimental test | | | ANCOVA |
|---|---|---|---|---|
| | STROOP TEST | Mean | SE | P value |
| Control | Baseline | 56.462 | 7.646 | 0.12 |
| | Post-experiment | 49.462 | 7.712 | |
| Non-winner | Baseline | 60.923 | 7.646 | 0.10 |
| | Post-experiment | 53.462 | 7.712 | |
| Winner | Baseline | 63.857 | 7.368 | 0.002** |
| | Post-experiment | 50.000 | 7.432 | |
| | BONFERRONI CORRECTION | Mean | SE | P value |
| Control | Baseline | 58.4829 | 7.1961 | 0.12 |
| | Post-experiment | 51.4829 | 7.6826 | |
| Non-winner | Baseline | 54.6095 | 7.3430 | 0.10 |
| | Post-experiment | 47.1479 | 7.8204 | |
| Winner | Baseline | 67.8428 | 6.9610 | 0.002** |
| | Post-experiment | 53.9857 | 7.4282 | |
| | FINGER TAPPING | Mean | SE | P value |
| Control | Baseline | 44.0000 | 4.1587 | 1.00 |
| | Post-experiment | 44.0000 | 4.1624 | |
| Non-winner | Baseline | 45.4615 | 4.1587 | 0.72 |
| | Post-experiment | 46.0769 | 4.1624 | |
| Winner | Baseline | 49.5000 | 4.0074 | 0.01* |
| | Post-experiment | 53.7143 | 4.0110 | |
| | BONFERRONI CORRECTION | Mean | SE | P value |
| Control | Baseline | 42.8591 | 4.0693 | 1.00 |
| | Post-experiment | 42.8591 | 4.0368 | |
| Non-winner | Baseline | 46.7950 | 4.0080 | 0.72 |
| | Post-experiment | 47.4104 | 3.9751 | |
| Winner | Baseline | 49.3212 | 3.8038 | 0.01* |
| | Post-experiment | 53.5355 | 3.7716 | |
| | 14-MWT | Mean | SE | P values |
| Control | Baseline | 29.6923 | 2.9600 | 0.67 |
| | Post-experiment | 30.3846 | 3.3732 | |
| Non-winner | Baseline | 36.7692 | 4.9822 | |
| | Post-experiment | 34.3846 | 4.9010 | |
| Winner | Baseline | 31.0000 | 1.8694 | |
| | Post-experiment | 29.7857 | 2.1360 | |
| | BONFERRONI CORRECTION | Mean | SE | P value |
| Control | Baseline | 3.3816 | 0.0652 | 0.75 |
| | Post-experiment | 3.3915 | 0.0676 | |
| Non-winner | Baseline | 3.4528 | 0.0659 | 0.02* |
| | Post-experiment | 3.3783 | 0.0682 | |
| Winner | Baseline | 3.4324 | 0.0627 | 0.11 |
| | Post-experiment | 3.3842 | 0.0650 | |

14-MWT = 14-meter Walk Test.

activation [16]. Preuschoff et al. [17] have shown that a contrast between winning and losing reveals significant activation of the subcortical circuitry including caudate, globus pallidus, thalamus and putamen as well as midbrain and cingulated gyrus. Our findings are not only in accordance with the latter report but further suggest that the practice of gaming, independently of wins and losses modifies favorably the dopaminergic circuitry in PD.

Dopaminergic neurotransmission plays an important role in sensory-motor integration, attention, incentive motivation and learning reinforcement behavior [17,18]. Dopamine activity at the nucleus accumbens, and more broadly the ventral striatal circuitry, has been shown to be involved in the so-called reward system [19]. In nigro-striatal motor control system, dopamine is present in a regular or relatively constant level, while in the cortico-mesolimbic system, dopamine and the positive reinforcement would be associated with phasic concentration spikes [20,21]. It has been suggested that both mechanisms participate in the control of nigro-striatal motor function and cortico-mesolimbic positive reinforcement, and a possible interaction between the two systems probably occurs [22]. Thus, motor-activating and reinforcement learning are probably both related to tonic and phasic dopamine signaling. In this context, selective actions of dopamine are unlikely. Studies about the effects of playing games on PD using brain imaging and also the chronic versus acute effects on cognition and motor function in PD are warranted.

Cognitive decline and dementia are important issues and have frequently been related to increased morbidity and mortality in PD [23]. Commonly, loss of executive function and other cognitive domains such as language and visuospatial skills occur and may remain undetected. The Stroop Color-Word Test requires frontal cortex inhibition and precociously detects impaired cognition, having been demonstrated in other conditions, such as malingering [24], and in cases of mild dementia versus depression [25]. Moreover, the Stroop test has been shown to correlate with functional disability in PD [26]. Previously, the dopaminergic modulation of prefrontal function has been demonstrated and it has been suggested that this is more evident in states of increased hypodopaminergic state function [27]. In this context, playing games would be more beneficial for patients in more advanced phases of disease and requiring more mental flexibility, frontal inhibition and executive function behavior which are essential for all vital abilities. Unfortunately, Non-Winners had initial non-significant better time in the Stroop test than winners. However, in this study, both groups improved executive function after gaming.

In this series most patients had depressive symptoms and this was not different among groups. Depressive symptoms were negatively correlated to motor skills before and after the experiment. The dopaminergic system plays a central role in mediating the effects of stress on motor function [28]. Furthermore, fluctuations of the motor system function are recognized to be influenced by anxiety, emotional issues and stress in PD. Attempts to explain the link between anxiety and motor fluctuation have claimed that hipokinesia would provoke anxiety and conversely it has been observed that emotional symptoms trigger off periods. Unwanted predictable wearing off and sudden unpredictable off periods are frequent and disabling symptoms in PD and need a better understanding. How the limbic system or more specifically, the cortico-mesolimbic system influences oscillations of the basal ganglia is not known. Interactions between the basal ganglia and cerebral cortex, and drive back have been shown to depend on dopamine and have oscillatory and phasic characteristics [29]. Local field potentials in the human basal ganglia have been demonstrated to be influenced by the behavioral relevance of external cues [30]. Taking this in consideration, the influence of playing games on the manifestation of fluctuations and off periods in PD patients should be of interest.

Limitations of this study must be acknowledged. First, a practice effect can not be excluded considering that tests were repeated within a short time. However, these conditions were similar for control and case groups. Other important consideration is that controls were kept seated and reading or watching television which are not specific tasks.

In summary, our results support a role for acute effects of gaming in ameliorating motor function and memory in PD. Further studies on this subject are in need to understand the role of gaming in PD.

## Acknowledgement

We wish to thank MCT/CNPq and FUNCAP for partial support of this study.
